# The Origins and Genetic Diversity of HIV-1: Evolutionary Insights and Global Health Perspectives

**DOI:** 10.3390/ijms262210909

**Published:** 2025-11-11

**Authors:** Ivailo Alexiev, Reneta Dimitrova

**Affiliations:** National Reference Confirmatory Laboratory of HIV, National Center of Infectious and Parasitic Diseases (NCIPD), 1504 Sofia, Bulgaria; naydenova.reneta@gmail.com

**Keywords:** HIV, zoonotic transmission, viral evolution, genetic diversity, molecular epidemiology

## Abstract

Human immunodeficiency virus (HIV), comprising two distinct types, HIV-1 and HIV-2, remains one of the most significant global health challenges, originating from multiple cross-species transmissions of simian immunodeficiency viruses (SIVs) in the early 20th century. This review traces the evolutionary trajectory of HIV from zoonotic spillover to its establishment as a global pandemic. HIV-1, the principal strain responsible for AIDS, emerged from SIVcpz in Central African chimpanzees, with phylogenetic evidence indicating initial human transmission between the 1920s and 1940s in present day Democratic Republic of Congo. The virus disseminated through colonial trade networks, reaching the Caribbean by the 1960s before establishing endemic transmission in North America and Europe. HIV’s extraordinary genetic diversity—driven by high mutation rates (~10^−5^ mutations per base per replication cycle) and frequent recombination events—has generated multiple groups, subtypes, and circulating recombinant forms (CRFs) with distinct epidemiological patterns. HIV-1 Group M, comprising subtypes A through L, accounts for over 95% of global infections, with subtype C predominating in sub-Saharan Africa and Asia, while subtype B dominates in Western Europe and North America. The extensive genetic heterogeneity of HIV significantly impacts diagnostic accuracy, antiretroviral therapy efficacy, and vaccine development, as subtypes exhibit differential biological properties, transmission efficiencies, and drug resistance profiles. Contemporary advances, including next-generation sequencing (NGS) for surveillance, broadly neutralizing antibodies for cross-subtype prevention and therapy, and long-acting antiretroviral formulations to improve adherence, have transformed HIV management and prevention strategies. NGS enables near real-time surveillance of drug resistance mutations and inference of transmission networks where it is available, although access and routine application remain uneven across regions. Broadly neutralizing antibodies demonstrate cross-subtype efficacy, while long-acting formulations have the potential to improve treatment adherence. This review synthesizes recent evidence and offers actionable recommendations to optimize clinical and public health responses—including the routine use of genotypic resistance testing where feasible, targeted use of phylogenetic analysis for outbreak investigation, and the development of region-specific diagnostic and treatment algorithms informed by local subtype prevalence. While the understanding of HIV’s evolutionary dynamics has substantially improved and remains essential, translating this knowledge into universally implemented intervention strategies remains a key challenge for achieving the UNAIDS 95-95-95 targets and the goal of ending AIDS as a public health threat by 2030.

## 1. Introduction

The discovery of human immunodeficiency virus (HIV) in the early 1980s marked a pivotal moment in modern medicine and public health. What began as isolated reports of unusual opportunistic infections among previously healthy individuals in the United States quickly evolved into recognition of a global pandemic that would fundamentally challenge our understanding of viral pathogenesis, immune function, and infectious disease control. The identification of HIV as the causative agent of acquired immunodeficiency syndrome (AIDS) represented both a remarkable scientific achievement and the beginning of an ongoing battle against one of the most genetically complex and evolutionarily adaptable pathogens known to science.

The ongoing evolution of HIV-1 continues to influence clinical management strategies in 2025. Given the 1.3 million new infections each year and 40.8 million people living with HIV globally [1], a comprehensive understanding of viral diversity is critical for effective and sustainable treatment approaches. Recent data demonstrate that 10–15% of new infections in sub-Saharan Africa involve drug-resistant strains [2], while non-B subtypes now account for >20% of new diagnoses in Western Europe and North America [3]—regions historically dominated by subtype B. These shifts directly impact diagnostic accuracy, with commercially available assays showing reduced sensitivity for divergent strains and treatment efficacy, as demonstrated by recent reports of virological failure in patients with subtype A6/A1 on long-acting injectable regimens [4].

The discovery of HIV was built upon earlier breakthroughs in retrovirology, particularly the identification of human T cell leukemia viruses (HTLV-1 and HTLV-2) in the late 1970s and early 1980s, which established that retroviruses could indeed cause human disease [5,6]. These foundational discoveries provided crucial technical and conceptual frameworks that enabled researchers to isolate and characterize HIV when the AIDS epidemic emerged rapidly. However, unlike HTLV, which is largely restricted to specific geographic regions and populations, HIV demonstrated an unprecedented scale of global dissemination and genetic diversification that would define the modern pandemic era.

The evolutionary story of HIV represents one of the most extensively documented examples of viral emergence, cross-species transmission, and pandemic spread in human history. Through sophisticated phylogenetic analyses, molecular clock studies, and recovery of viral sequences from historical samples, the evolutionary pathway of HIV has been reconstructed from its origins in Central African primates to its emergence as a global pandemic. This remarkable scientific detective work has revealed that HIV-1, the predominant pandemic strain, likely emerged in the early 20th century through cross-species transmission from chimpanzees, spreading through colonial trade networks and achieving global dissemination driven by patterns of human migration, urbanization, and social change characteristic of the latter half of the 20th century.

Understanding the origins of HIV, its evolution, and global spread is not merely of historical interest, but it also provides essential insights for contemporary public health challenges and future pandemic preparedness. The extraordinary genetic diversity of the virus, driven by high mutation rates and frequent recombination, continues to present significant challenges for diagnostics, treatment, and vaccine development. Different HIV subtypes and circulating recombinant forms exhibit distinct biological properties, geographic distributions, and clinical characteristics that directly impact disease management strategies. Moreover, the continued evolution of HIV under selective pressures from host immune responses and antiretroviral therapy necessitates ongoing molecular surveillance and adaptive public health approaches. This review synthesizes current knowledge of the zoonotic origins of HIV, evolutionary mechanisms, global dissemination patterns, and the continuing implications of viral genetic diversity for HIV prevention, treatment, and potential cure strategies.

## 2. Discovery of HIV and Its Link to AIDS

In the early 1980s, a novel syndrome marked by severe opportunistic infections, malignancies, and immune suppression emerged, rapidly spreading and raising global health concerns. The first U.S. cases, involving Pneumocystis jirovecii pneumonia and Kaposi sarcoma, were reported in 1981 [7]. Epidemiological patterns prompted urgent investigation. This culminated in 1983 with the isolation of a new virus, initially termed lymphadenopathy-associated virus (LAV) [8] by one group, and human T-lymphotropic virus type III (HTLV-III) [9] by others. In 1986, it was unified under the name human immunodeficiency virus (HIV).

HIV spreads primarily through sexual contact, blood exposure (including non-sterile medical equipment, transfusions, and shared needles) [10], and vertical transmission from mother to child during pregnancy, delivery, or breastfeeding. Without prophylaxis, vertical transmission rates may reach 45%, but with modern antiretroviral interventions, the risk drops below 2% [11]. The virus, a member of the Retroviridae family (genus Lentivirus), contains structural genes (gag, pol, env) encoding core proteins, enzymes, and envelope glycoproteins, as well as regulatory genes (tat, rev, nef, vif, vpr, vpu) that promote replication and immune evasion [12].

The HIV lifecycle begins with attachment to CD4+ T lymphocytes via gp120 and co-receptors (CCR5 or CXCR4), followed by fusion and entry. Reverse transcriptase converts RNA to DNA, which is inserted into the host genome by integrase. Host machinery then produces viral components that assemble and bud from the cell, leading to CD4+ depletion, immune dysfunction, and susceptibility to opportunistic infections and cancers. Infection progresses through acute HIV (2–4 weeks post-exposure, with fever, rash, and lymphadenopathy) [13], chronic latency (often asymptomatic) [14], and AIDS, which is defined by CD4+ < 200 cells/μL or AIDS-defining illnesses [15].

Insights into HIV’s molecular biology enabled antiretroviral therapy (ART), which has greatly improved prognosis and life expectancy. However, global control remains incomplete, demanding sustained prevention efforts and vaccine development.

## 3. Origin of HIV

HIV-1 exhibits significant genetic diversity driven by two primary molecular mechanisms: high mutation rate and recombination. These enable rapid evolution, facilitating immune evasion and resistance to antiretroviral therapy, which complicates disease control [16,17]. Multiple zoonotic events involving simian immunodeficiency viruses (SIVs) underlie the origin of HIV-1 subtypes, with Groups M and N originating from SIVcpz (chimpanzees), while groups O and P are related to SIVgor (gorillas), resulting in genetically distinct viral lineages with unique epidemiological and clinical profiles [18,19].

The virus continues to evolve under host immune pressure, as cytotoxic T lymphocytes and neutralizing antibodies select for the emergence of escape mutants, while HIV employs counterstrategies like Nef-mediated immune evasion and altered glycosylation. Subtype variation influences disease progression, resistance patterns, and vaccine responsiveness, with certain subtypes differing in CD4+ T cell decline rates and harboring specific resistance mutations, requiring tailored treatment approaches [20,21]. The global heterogeneity of HIV-1 subtypes and their clinical implications necessitate international cooperation in genomic surveillance and research. Ongoing virological studies and the development of broad-spectrum therapies and vaccines remain critical for addressing the evolutionary adaptability of HIV-1 [3,22].

HIV originated from SIVs, a group of lentiviruses infecting over 40 non-human primate (NHP) species across sub-Saharan Africa [18]. In natural hosts like African green monkeys, sooty mangabeys (*Cercocebus atys*), and mandrills, SIV causes little to no disease due to long-term co-evolution and viral attenuation [18,23]. Upon crossing into humans, however, SIV adapts to its new host, giving rise to HIV-1 (from SIVcpz) and HIV-2 (from SIVsmm), which are both capable of causing AIDS [20,24]. These transmissions likely occurred through direct exposure to infected blood or bodily fluids during hunting and handling of bushmeat, particularly under conditions of mucosal injury [25]. Crucially, HIV did not originate from a single spillover event, but rather from multiple independent zoonotic transmissions, resulting in the emergence of distinct groups: HIV-1 (M, N, O, P) and HIV-2 (A–H). Among these, only group M (HIV-1) and groups A and B (HIV-2) have established sustained human-to-human transmission [19].

HIV-1, the most prevalent and virulent form, originated from SIVcpz in central African chimpanzees, likely via exposure to infected blood during bushmeat practices [18,26]. Keele et al. [26] provided molecular evidence by identifying SIVcpz in wild chimpanzees across Central Africa, while further phylogeographic research by Worobey et al. [27] traced the emergence of HIV-1 group M, the pandemic strain to chimpanzees in southeastern Cameroon, with subsequent spread via colonial trade routes to Kinshasa (then Léopoldville), which became the epicenter of the epidemic. Viral adaptation involved mutations in env and vpu that increased infectivity and neutralized host restriction factors like tetherin (BST-2) [20], while socio-behavioral factors including urbanization, unsafe medical practices, and changing sexual networks amplified the spread during the 20th century.

In contrast, HIV-2 represents a less transmissible and less virulent form that emerged from SIVsmm in sooty mangabeys, which are endemic to West Africa [24]. Although HIV-2 has spread beyond the region, it remains largely confined to West Africa. The genetic link between SIVsmm and HIV-2 was first demonstrated by Hirsch et al. [28] confirming an independent zoonotic origin; and while most spillovers have been self-limited, Cantlay et al. [25] documented multiple recent cross-species transmissions, emphasizing the ongoing risk and the need for targeted surveillance. Clinically, HIV-2 is associated with slower disease progression, lower viral loads, and prolonged asymptomatic phases, with infected individuals maintaining higher CD4+ T cell counts than those with HIV-1 [29]. These characteristics are attributed to reduced viral replication and different immune evasion tactics; though importantly, HIV-2 shows natural resistance to several non-nucleoside reverse transcriptase inhibitors (NNRTIs), requiring alternative ART regimens and precise therapeutic strategies [30].

While HIV-1 Group M drives the global pandemic, HIV-2 remains a significant regional health concern. Their independent zoonotic origins from different SIV strains have resulted in fundamentally distinct clinical and epidemiological profiles. Table 1 provides a clear comparative summary of the key characteristics of HIV-1 Group M and HIV-2, illustrating the differences in virulence, transmission efficiency, and natural drug resistance that mandate varied clinical management approaches.

Understanding the origins of HIV underscores the importance of monitoring human–primate interactions with enhanced surveillance and viral discovery in high-risk regions, being essential to predict and prevent future zoonotic pandemics.

## 4. Timeline of the Zoonotic Transition and Factors Facilitating the Spread of HIV

The earliest genetically confirmed HIV-1 sample, ZR59, was obtained in 1959 from a person in Kinshasa, Democratic Republic of Congo [31]. While ZR59 does not mark the zoonotic origin itself, it provides critical evidence for early HIV-1 presence in humans and helps reconstruct the evolutionary trajectory of the virus. Phylogenetic comparisons show significant genetic diversity even at that stage, indicating that spread and diversification were already underway. An additional historical sample, a 1960 lymph node biopsy from Kinshasa, demonstrates that HIV-1 group M was already genetically diverse by that time, implying that the SIV-to-HIV transition likely occurred decades earlier [27]. Molecular clock analyses estimate this zoonotic event between the 1920s and 1940s, based on divergence times and early viral lineages, with Rambaut et al. [32] estimating that the most recent common ancestor of HIV-1 group M emerged in the early 20th century, followed by rapid diversification.

Phylogenetic and phylogeographic studies confirm that HIV-1 group M likely originated in southeastern Cameroon and reached Kinshasa through human mobility [26]. This suggests a complex process of viral adaptation embedded within broader evolutionary and social dynamics. Worobey et al. [27] used phylogenetic methods to show that HIV-1 likely spread from Kinshasa to other African regions and globally, facilitated by a well-developed railway network that linked Kinshasa with much of Central Africa, enabling viral dissemination to nearby population centers and eventually to the Caribbean. Human mobility, especially labor migration and trade, played a central role in spreading HIV, with studies emphasizing that colonial era infrastructural changes contributed significantly to early dissemination [22].

Beyond transportation, multiple interconnected factors accelerated HIV transmission during the early 20th century. Pepin [33] notes that mass vaccination programs during the colonial period likely accelerated transmission through the reuse of unsterilized syringes, while urbanization, population growth, and evolving sexual networks further enabled viral spread. Historical data analyzed by de Sousa et al. [34] identify a high prevalence of genital ulcerative diseases (GUDs) in the early 20th century, which increased susceptibility to blood-borne transmission and accelerated HIV spread among humans. The sex industry and unsafe medical practices also contributed significantly to early viral dissemination.

After HIV-1 group M became established, its international spread followed migration and transport routes, with genetic evidence showing that subtype B reached Haiti in the 1960s, likely via Haitian workers returning from Congo, from where the virus spread to the United States [35], marking a critical step in global transmission. By the early 1970s, HIV-1 was present in North America, and the expansion of air travel later in the century enabled rapid spread to Europe, Asia, and beyond [27]. This period illustrates how biological, social, and infrastructural forces jointly shaped the establishment of a global pandemic, with globalization, labor migration, and the movement of military personnel further accelerating viral spread beyond Africa. Junqueira et al. [36] confirm, through genetic evidence, that migration from Central Africa seeded HIV-1 in the Caribbean, later reaching the U.S. and Europe, allowing HIV to enter high-income countries where it initially spread within marginalized populations before being widely recognized.

Structural inequalities significantly exacerbated HIV transmission patterns throughout this period. UNAIDS [37] and Garrett [38] emphasize how under-resourced health systems, weak diagnostics, and limited public health measures perpetuated viral spread during and after the colonial era; and conversely, poverty and poor healthcare access created additional vulnerabilities. Gender dynamics also profoundly influence transmission, with Quinn and Overbaugh [39] noting that women in sub-Saharan Africa are disproportionately affected due to limited healthcare access, socio-economic dependence, and reduced autonomy in sexual and reproductive decision-making. Cultural norms, economic pressures, and lack of education often force women into high-risk situations, including the sex industry, which remains a major transmission route.

## 5. Evolution of HIV

HIV, a member of the Retroviridae family, replicates by integrating its RNA genome into host DNA via the enzyme reverse transcriptase. This process enables lifelong persistence and complicates eradication, as the virus can evade immune detection and develop antiretroviral resistance [40]. The concept of reverse transcription, proposed by Howard Temin, underpins our understanding of retroviral replication [41], with integration into host DNA allowing HIV to persist despite ART, necessitating lifelong treatment [42]. Curative strategies, including CRISPR/Cas9, remain complex due to latent viral reservoirs unaffected by standard ART [43], though preclinical studies suggest potential for excising of latent genomes [44].

The defining feature of HIV is its extraordinarily high mutation rate, stemming from the lack of proofreading capacity in reverse transcriptase, which leads to extensive genetic variability and immune evasion [16]. Roberts et al. [45] estimate a mutation rate of ~3 × 10^−5^ per base per cycle, one of the highest observed among viruses, resulting in quasispecies within individuals. These closely related variants complicate vaccine development and therapeutic strategies [21], fostering drug resistance as mutations alter viral proteins targeted by ART, requiring combination therapies [46]. Moreover, env gene mutations enable escape from neutralizing antibodies, further hindering vaccine efficacy [47].

**Figure 1:**
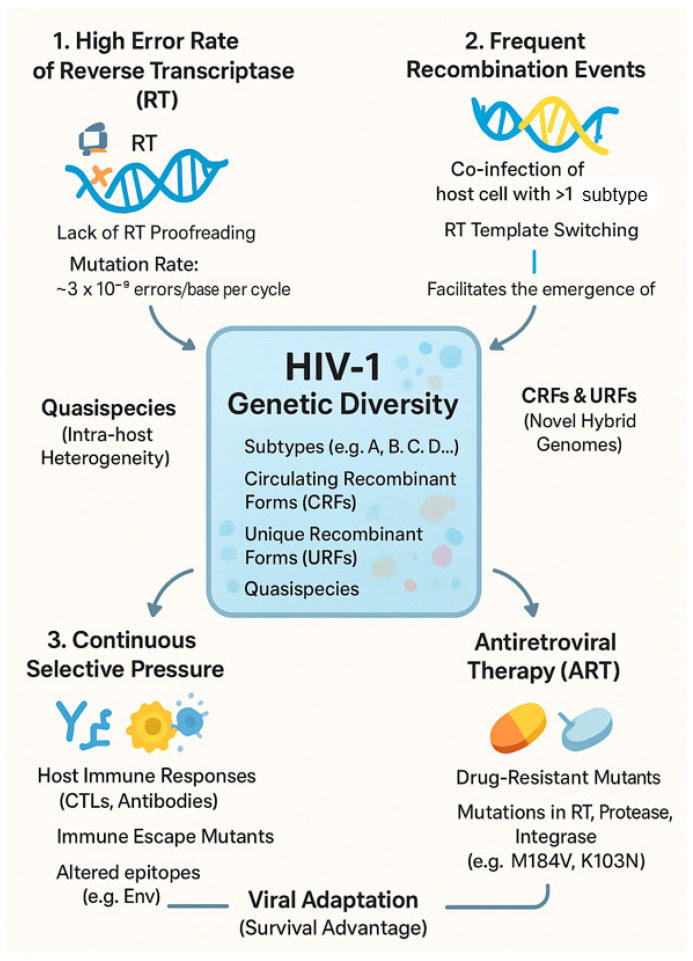
Molecular and selective forces driving the genetic diversity and evolution of HIV-1.

The remarkable genetic heterogeneity that defines the AIDS pandemic is a direct consequence of the unique interplay between the virus’s replication cycle and the pressures it faces within and between hosts. This rapid evolution, which creates a continuous emergence of new variants, is driven by three primary and reinforcing forces: the high error rate of reverse transcriptase, frequent recombination events, and intense selective pressures from both the host immune system and antiretroviral drugs. The collective action of these drivers allows HIV-1 to rapidly adapt, resulting in the complex landscape of subtypes, circulating recombinant forms (CRFs), unique recombinant forms (URFs), and quasispecies observed globally. Figure 1 provides a schematic overview of how these molecular and selective forces converge to generate the extensive genetic diversity of HIV-1.

This extraordinary genetic diversity arises from the high mutation rate of HIV, recombination, and phylogenetic complexity, posing challenges for diagnostics, treatment, and vaccines that must be understood to control global transmission [3,17]. HIV mutates nearly 1000 times faster than DNA viruses, with reverse transcriptase lacking error correction and generating frequent nucleotide substitutions, insertions, and deletions [45]. These mutations promote adaptation to immune pressure and ART, while the host enzyme APOBEC3G induces G-to-A hypermutations during reverse transcription, sometimes inactivating the virus [21]. However, the Vif protein of HIV counters APOBEC3G by targeting it for degradation, exemplifying a host–virus arms race [17,21].

HIV-1, the pandemic strain, includes four groups: Group M (Major) accounting for over 95% of cases, Group O (Outlier), Group N (Non-M, Non-O), and Group P [17]. Group M contains subtypes A, B, C, D, F, G, H, J, K, L, with subtype B dominating in North America and Europe, subtype C prevailing in Southern Africa and India, and subtypes A, D, and various CRFs circulating in Africa. HIV-2, less transmissible and virulent, is largely confined to West Africa and consists of subtypes A–H [48]. This genetic plasticity complicates accurate diagnosis and effective vaccine design. Constant mutation and recombination necessitate diagnostic tools capable of detecting diverse forms, resistance mutations demand adaptive ART regimens, and subtype variation hinders vaccine development because protective immunity may not extend across all variants [16,17].

Beyond mutation and recombination, selective pressures from the host immune system and antiretroviral therapy play a major role in shaping the diversity of HIV-1. Cytotoxic T lymphocytes (CTLs) and neutralizing antibodies exert strong immune pressure, with CTLs identifying and eliminating infected cells by recognizing viral peptides presented via MHC molecules. HIV-1 can accumulate mutations in these regions—an immune escape mechanism that impairs CTL recognition and enables viral persistence [37]. Neutralizing antibodies target epitopes on the viral envelope glycoproteins sgp120 and gp41, and mutations in these regions help the virus evade antibody-mediated neutralization and contribute to genetic heterogeneity. The envelope glycoproteins are among the most variable regions in the HIV-1 genome and undergo rapid evolution under immune pressure [38], complicating vaccine development and requiring immunogens capable of eliciting broad immune responses.

ART represents another major selective force, targeting various stages of the viral lifecycle, such as reverse transcription, integration, and proteolytic processing. The high mutation rate of HIV-1 enables the emergence of resistant variants, especially when ART fails to fully suppress replication [39]. Resistance mutations, such as M184V and K103N in reverse transcriptase, confer resistance to NRTIs and NNRTIs, depending on the regimen used. The evolution of resistant strains threatens the long-term efficacy of ART, emphasizing the importance of treatment adherence and the development of new agents. Since some resistant strains are transmissible, it leads to primary resistance in treatment-naïve individuals and highlights the need for surveillance programs to monitor drug-resistant HIV and adapt therapeutic strategies accordingly [40].

HIV-1 subtypes differ significantly in genetic, biological, and clinical traits, which affect their transmission, pathogenicity, and immune interactions. A key distinction among subtypes lies in the env gene, which encodes sgp120 and gp41—glycoproteins involved in viral entry via CD4 and co-receptors CCR5 or CXCR4. Variations in env affect viral tropism, with some subtypes predominantly using CCR5 (R5-tropic), which is common in early infection, while others may evolve to use CXCR4 (X4-tropic), which is often linked to disease progression [49]. Subtype C is the most prevalent globally and exhibits greater env diversity than subtype B [42], potentially enhancing immune evasion and adaptability to different host populations. Variability in the *env* also impacts antibody recognition, as sgp120 includes multiple epitopes targeted by neutralizing antibodies, and subtype-specific differences influence the effectiveness of immune responses. Some subtypes contain highly immunogenic epitopes, while others present cryptic or highly variable ones that impede antibody binding [38], complicating vaccine design and necessitating broad, cross-subtype immune responses.

Understanding the evolutionary dynamics of HIV is critical for public health, with ongoing molecular surveillance, research on mutation and recombination, and innovative therapeutic strategies being essential for containing the epidemic.

## 6. Biological Properties, Clinical Manifestations, and Public Health Significance of HIV-1 Subtypes

Beyond genetic variation, HIV-1 subtypes differ significantly in biological traits like replicative capacity and transmission efficiency, with important clinical and public health implications. Replicative capacity refers to how efficiently the virus replicates in host cells, which may vary by subtype, as some subtypes replicate more efficiently in macrophages or T cells due to differences in viral proteins or regulatory sequences [43]. This impacts viral load, disease progression, and overall fitness. Transmission efficiency also varies among subtypes, with subtype C, the most prevalent globally, being linked to higher heterosexual transmission rates [44]. Factors like elevated viral loads in genital secretions, greater stability, and improved mucosal interaction may explain this, while subtype C also exhibits stronger affinity for dendritic cells, enhancing sexual transmission [16]. Another differing trait is the ability to establish latent reservoirs, which allow HIV-1 to persist despite ART, with some subtypes potentially being more efficient at latency due to unique integration patterns or interactions with host factors [45]. These differences are crucial for developing latency-targeting therapies.

Subtypes also differ in clinical progression and drug resistance patterns, with some, like subtype D, being linked to faster AIDS progression than subtype A, while subtype C may progress more slowly in some populations [50]. However, findings are mixed, suggesting roles for both viral and host immune factors. Immune escape capacity may underlie these trends, as subtype D shows higher immune escape potential, possibly accelerating disease [51], while subtype C may elicit stronger immune responses, contributing to slower progression. Host factors such as HLA type and immune activation levels also modulate clinical outcomes. Drug resistance varies significantly across subtypes, with subtype B, which dominates in North America and Europe, being well studied; and many resistance mutations were first identified in this group [46]. Non-B subtypes, more common in resource-limited regions, may show distinct resistance patterns due to genetic differences. For instance, subtype C has a lower genetic barrier to resistance against some NNRTIs like nevirapine, allowing mutations to develop more rapidly, which may limit treatment efficacy [52].

HIV-1 Subtype A6/A1 has been associated with a potentially increased risk of virological failure when treated with long-acting injectable regimens such as cabotegravir and rilpivirine. Pre-treatment genotyping is becoming increasingly important for personalized therapy selection. Recent trials, particularly SOLAR and ATLAS-2M, as well as the analysis by Cutrell et al. [4], clearly identified subtype A6/A1 as a risk factor for virological failure. These findings demonstrate that genetic variations in subtype A6/A1 may reduce susceptibility to long-acting regimens through specific mutations in integrase and reverse transcriptase genes, potentially impairing treatment effectiveness and raising the risk of resistance. This highlights the critical importance of genotypic resistance testing and close virological monitoring (or alternative regimens) in patients with A6/A1 on long-acting ART. These subtype-related differences underscore the need for personalized ART strategies, tailored to viral and host genetic contexts, to improve the management of HIV. 1. Differences among HIV-1 subtypes have critical public health implications, particularly for diagnostics, treatment, and prevention. The genetic diversity of the virus complicates the development of diagnostic tests, which must detect all subtypes and circulating recombinant forms (CRFs), as tests targeting conserved genome regions may miss highly divergent strains, risking false negatives [53]. Antiretroviral drug and vaccine development must also account for subtype variability to ensure global efficacy. Variations in genes like env can alter key epitopes, affecting drug susceptibility and immune recognition, thereby complicating efforts to create broadly effective vaccines. Understanding subtype-specific features is essential for managing the global HIV/AIDS epidemic, with advances in next-generation sequencing (NGS) having transformed HIV research by enabling real-time monitoring of viral evolution. Integrating whole-genome NGS with computational and functional analyses offers insights into mechanisms driving diversity, supporting the development of more accurate diagnostics, personalized treatment regimens, and vaccines tailored to the unique challenges posed by different HIV-1 subtypes.

## 7. HIV-1 Recombination

Recombination is a central driver of HIV genetic diversity and evolution, occurring when two distinct viral genomes infect the same cell and exchange genetic material during reverse transcription. This process generates hybrid genomes with novel biological traits and is especially frequent in regions of high subtype diversity, such as sub-Saharan Africa [3,17]. It occurs as a reverse transcriptase (RT), which lacks proofreading and switches templates between co-packaged RNAs, producing recombinant DNA-containing sequences from both viruses [54]. Recombination is particularly common in homologous regions, notably the env gene-encoding envelope glycoproteins [55].

This mechanism drives the emergence of circulating recombinant forms (CRFs) and unique recombinant forms (URFs). CRFs spread widely in areas where multiple subtypes co-circulate, for example, CRF02_AG in West Africa and CRF01_AE in Southeast Asia [3,21], and often display distinct transmission dynamics and resistance patterns, complicating diagnosis and therapy. URFs, in contrast, are localized variants most common in regions with extensive subtype diversity, such as East and Central Africa. Their continuous emergence underscores the dynamics of HIV evolution, reinforcing the importance of monitoring recombination for epidemic control.

The continuous emergence of CRFs and URFs, coupled with the long-standing geographic segregation of established subtypes, results in a complex, heterogeneous global epidemic. Table 2 summarizes the current global prevalence, dominant geographic distribution, and key biological or clinical traits of the most significant HIV-1 Group M subtypes and circulating recombinant forms, highlighting the variability that is crucial for targeted surveillance and intervention strategies.

The global distribution of subtypes and CRFs is shaped by historical, social, and biological factors, and must be understood to optimize prevention, treatment, and surveillance [3,56]. Sub-Saharan Africa, the epicenter of the HIV/AIDS pandemic, exhibits exceptional viral diversity, with nearly all subtypes and numerous CRFs. Subtype C dominates, accounting for ~50% of global infections, particularly in Southern and Eastern Africa, likely due to high transmission efficiency and elevated genital viral loads [3,22]. Subtype A is common in Eastern Africa, while CRF02_AG predominates in West and Central Africa (e.g., Nigeria, Cameroon) [3]. This diversity reflects both the long-standing presence of HIV and frequent coinfections that facilitate recombination [57], with variability in env posing particular challenges for diagnostics and vaccine design.

In Asia, HIV-1 diversity mirrors multiple introductions and transmission routes. CRF01_AE dominates Southeast Asia (Thailand, Vietnam, Cambodia), associated with heterosexual transmission and injection drug use [3,58]. In East Asia, subtype B is prevalent in Japan and South Korea, primarily among men who have sex with men and people who inject drugs [59], while China harbors major recombinants such as CRF07_BC and CRF08_BC, originating from B/C recombination and spreading among PWID and populations exposed to unsafe blood practices. By contrast, subtype B historically dominated North America, Western Europe, and Australia, linked to men who have sex with men and injection drug use [3,60]. Yet migration and globalization have increased the prevalence of non-B subtypes and CRFs—particularly C, CRF02_AG, and CRF01_AE—in urban immigrant communities [3,61], with non-B strains now representing a growing fraction of new US infections, especially among African and Asian immigrants [62].

The prevalence of subtype and CRF shifts over time due to migration, travel, and behavioral changes, introducing new strains and facilitating further recombination, as observed with the rise in non-B subtypes in Europe and North America [3,63]. Public health interventions also influence trends: while antiretroviral therapy reduces prevalence, it may alter subtype distribution, with transmissible or drug-resistant variants gaining relative prominence [64]. This global diversity poses challenges for diagnostics, treatment, and prevention, as assays targeting conserved regions risk false negatives with divergent strains [53], and therapeutic and vaccine strategies must account for extensive viral variability.

Evidence regarding HIV recombinants in PrEP breakthrough infections remains limited but emerging evidence suggests that HIV recombinants may play a role in PrEP breakthrough infections, though comprehensive data remain limited. Preliminary surveillance reports indicate that CRFs and URFs are increasingly detected among PrEP users who acquire HIV, particularly in regions with high subtype diversity. Some recombinants may harbor polymorphisms affecting drug susceptibility, potentially impacting PrEP efficacy. This highlights the importance of continued molecular surveillance of breakthrough infections to better understand the role of viral diversity in PrEP effectiveness and inform future prevention strategies.

## 8. Global Spread of HIV-1

HIV-1 group M is the main strain driving the global AIDS pandemic, likely spreading from Kinshasa, Democratic Republic of the Congo (DRC) through trade routes and migrating to the Caribbean, the US, and Europe during the mid-20th century. Phylogenetic studies highlight key transport routes, like the DRC railway, as critical to its spread [22]. The virus reached Haiti in the 1960s via workers returning from Congo, then spread to the US and Europe, with urban centers such as New York, San Francisco, London, and Paris becoming early epidemic hotspots shaped by migration, socio-economic factors, and health infrastructure. Subtype B, which is dominant in the Americas and Europe, originated in the Caribbean, while subtype C, the most common globally found, spread through labor migration in Southern Africa [19,48]. In Asia, CRF01_AE entered Thailand in the late 1980s, spreading rapidly among high-risk groups [65], with increasing global travel and trade further facilitating the worldwide spread of HIV.

Armed conflicts and displacement often increase HIV transmission by disrupting health systems and social structures, creating conditions that amplify viral spread. For example, HIV prevalence more than doubled during the civil war in Guinea-Bissau, and Uganda experienced spikes in incidence during unrest [66]. In the DRC and other sub-Saharan African countries, prolonged conflict has hindered prevention and treatment, while refugee and military movements have spread HIV, with factors like unsafe blood transfusions and collapsed health services worsening the crisis [66]. The 2022 Russia– Ukraine war raised concerns over increased HIV risk due to displaced populations and disrupted treatment access, as Ukraine had 260,000 people living with HIV before the war, with many now facing treatment challenges amid displacement and increased vulnerability to sexual violence [67]. Economic migration also contributes significantly, with labor migration between Nepal and India being linked to higher transmission rates via expanded commercial sex work and limited access to preventive measures [68]. Overall, conflicts and migration, whether forced or economic, promote HIV spread by weakening health services and increasing population vulnerability, demonstrating how social disruption amplifies biological factors driving the pandemic.

## 9. Implications of HIV-1 Genetic Diversity: Epidemiological Surveillance, Strategies, and Perspectives

The extensive genetic diversity of HIV-1, manifested in multiple subtypes, CRFs, and URFs, profoundly influences molecular epidemiology. This variability affects diagnostic accuracy, therapeutic efficacy, vaccine development, and public health surveillance, while presenting both challenges and opportunities for controlling the global HIV/AIDS pandemic [3,48].

### 9.1. Diagnostic Challenges

The pronounced heterogeneity of HIV-1, particularly in the envelope (env) and capsid (gag) genes, complicates the development of universal diagnostic tools. Diagnostic assays, including nucleic acid amplification tests and immunological assays (ELISA, Western blot, etc.), must reliably detect all known subtypes, CRFs, and URFs. However, assays that target conserved genomic regions may fail to recognize highly divergent variants, leading to false-negative results. This limitation is critical in regions where non-B subtypes and CRFs predominate, as many diagnostic platforms were initially optimized for subtype B, which is the most common lineage in North America and Europe [3]. Delayed diagnosis not only accelerates disease progression but also increases the risk of onward transmission. The continual emergence of new recombinant forms further underscores the need for regular updates to diagnostic platforms [69].

Traditional antibody-based methods such as ELISA and Western blot remain unreliable for detecting newly emerging subtypes, particularly during acute infection when antibody titers are low [70]. While rapid diagnostic tests have expanded access, their sensitivity and specificity vary substantially across variants [71].

### 9.2. Treatment and Drug Resistance

Genetic diversity across HIV-1 subtypes may influence ART effectiveness. Natural polymorphisms in reverse transcriptase, protease, and integrase genes may affect drug susceptibility, while recombination generates variants with complex and sometimes unpredictable resistance profiles [72,73]. Non-B subtypes, which dominate in Africa and Asia, exhibit distinct resistance patterns, acquiring mutations at different frequencies compared to subtype B [74]. These differences complicate treatment strategies and highlight the importance of global resistance monitoring, especially in resource-limited regions where surveillance capacity is often weakest. World Health Organization (WHO) prioritizes routine resistance surveillance to inform ART guidelines and prevent the spread of resistant strains [75].

New therapeutic approaches aim to counteract these challenges. Personalized ART strategies, long-acting injectable formulations, and broadly neutralizing antibodies (bNAbs) offer promising avenues for maintaining viral suppression while reducing the likelihood of resistance development [76].

### 9.3. Vaccine Development Challenges

Vaccine development has been hindered by HIV-1’s extraordinary genetic variability, driven by the lack of proofreading activity in reverse transcriptase and frequent recombination events [77]. Antigenic diversity is particularly pronounced in viral envelope glycoproteins gp120 and gp41, which are primary immune targets but display high conformational flexibility and variability across subtypes [78]. As a result, most vaccine candidates developed for subtype B populations, such as the VAX003 and VAX004 trials, have shown limited or no efficacy in regions where non-B subtypes predominate [79]. Innovative approaches attempt to circumvent these barriers. Mosaic antigens designed by combining conserved epitopes from multiple subtypes [80] and strategies focusing on highly conserved viral regions [81] aim to elicit broader, cross-clade immune responses. The recent success of mRNA vaccine technology against SARS-CoV-2 has renewed optimism [82]. In parallel, broadly neutralizing antibodies (bNAbs) targeting conserved envelope regions can block infection irrespective of subtype; however, the emergence of resistant mutations necessitates adaptive, combination-based prophylactic approaches [83]. Early HIV vaccine trials utilizing these novel technologies are already demonstrating the ability to elicit robust immune responses [84].

Despite these historical barriers, recent advancements across multiple immunological platforms have significantly renewed the trajectory of HIV-1 vaccinology and prevention. Foremost among these are novel immunization technologies, particularly mRNA platforms, which have rapidly advanced candidate vaccines like Moderna’s mRNA-1644 and mRNA-1574. These constructs leverage germline-targeting and conserved epitope strategies [84], successfully demonstrating the concept of eliciting VRC01-class precursor antibodies in up to 80% of participants in trials like IAVI G002 and G003 [85]. Complementing active immunization efforts, passive administration of bNAbs represents a mature, subtype-agnostic prophylactic strategy. Combination bNAb therapy, such as VRC01LS and 3BNC117-LS, successfully achieved durable six-month protection across diverse clades, including subtypes B, C, and CRF01_AE, in large-scale efficacy trials like HVTN 704/HPTN 085 [86,87]. Finally, while the pivotal HVTN 705/Mosaico trial for mosaic immunogens was discontinued, retrospective analysis has yielded crucial mechanistic insights (40% efficacy against certain clade C infections in related studies). These findings are instrumental in refining next-generation mosaic designs aimed at enhancing coverage and potency [76]. Collectively, these multi-pronged strategies are driving the field toward effective, globally relevant prevention tools.

### 9.4. Epidemiological Surveillance

Genetic diversity poses ongoing challenges for epidemiological surveillance. Mapping the distribution of subtypes is essential for understanding transmission dynamics, identifying high-risk groups, and monitoring emerging variants [88]. Phylogenetic analysis and molecular clock methods enable reconstruction of transmission networks and detection of rapidly expanding clusters [89]. Continuous viral evolution, through mutation and recombination, generates novel subtypes and CRFs with unique epidemiological traits. Recently described recombinant forms in West Africa and Southeast Asia highlight the need for constant global monitoring [48].

### 9.5. Research Priorities and Cure Strategies

Understanding the evolutionary origins and continuing diversification of HIV provides important insights for improving clinical and public health strategies. Cross-species transmission events that led to the emergence of HIV-1 and HIV-2 illustrate the remarkable adaptability of the virus and its capacity to exploit new cellular and ecological niches [18]. Likewise, ongoing within-host evolution—driven by high mutation and recombination rates, selective drug pressure, and host immune responses—continuously shapes viral characteristics that influence diagnostic accuracy, therapeutic response, and vaccine design.

Incorporating evolutionary and molecular perspectives into surveillance and treatment frameworks can enhance the prediction of clinical outcomes and support the development of adaptive intervention strategies [90]. For example, genomic surveillance helps identify emerging resistance mutations before treatment failure occurs, while phylogenetic analyses of recombinant forms contribute to mapping transmission clusters and refining prevention efforts. A better understanding of conserved viral regions also informs the design of broadly neutralizing antibodies and cross-subtype diagnostic assays. These concepts highlight that knowledge of HIV evolution is essential for achieving more effective and sustainable epidemic control.

Despite substantial progress in molecular characterization, the biological implications of subtype differences remain incompletely understood. Subtype C, responsible for nearly 50% of global infections, exhibits enhanced replication in macrophages, potentially facilitating transmission [91]. By contrast, subtype D has been associated with more rapid disease progression, possibly due to superior immune evasion mechanisms [50]. Further elucidating these functional differences remains a research priority.

Current curative strategies aim to eliminate persistent viral reservoirs, which remain the primary barrier to eradication. Approaches include the following:

- Gene editing technologies (CRISPR/Cas9, TALENs) to disrupt proviral DNA;
- Latency-targeting interventions, such as “Shock and Kill” (reactivating and clearing latent virus) and “Block and Lock” (permanently silencing proviral transcription);
- Immunotherapies, including therapeutic vaccines and bNAbs, to boost host immunity;
- Cellular therapies, most notably hematopoietic stem cell transplantation, have achieved rare cases of functional cure [92].

Identifying the most effective combination of these strategies remains an urgent area of investigation.

### 9.6. Global Health Implications

The global health implications of HIV-1 diversity are profound. Strengthening surveillance systems is critical for monitoring evolving molecular epidemiology, with NGS emerging as a transformative tool for genome-wide characterization [93]. Such platforms can track transmission clusters, identify emerging CRFs, and monitor drug-resistant strains in real time.

Equally important is improving education and awareness, as the implications of HIV-1 diversity are often poorly understood by healthcare providers and policymakers. Community-based interventions promoting pre-exposure prophylaxis (PrEP) and treatment as prevention (TasP) remain essential, particularly for high-risk populations.

Ultimately, sustainable epidemic control will require addressing structural determinants such as poverty, inequality, and limited healthcare access [94]. Achieving the UNAIDS 95-95-95 targets depends not only on scientific advances but also on expanding access to healthcare, removing barriers to therapy, and integrating social support systems. Confronting the challenges posed by HIV-1 diversity thus demands a multifaceted strategy that combines cutting-edge science with effective public health interventions. Global collaboration across disciplines and regions offers the best hope for reducing the burden of HIV/AIDS worldwide.

## 10. Conclusions

The origin and evolution of HIV is well documented through genetic, phylogenetic, and historical research, revealing that the virus likely originated from simian immunodeficiency virus (SIV) in primates and crossed into humans in the early 20th century. Its global spread results from a complex interplay of biological, social, and historical factors that continue to shape the pandemic today. Genetic studies of SIV and its cross-species transmission provide the foundation for understanding the zoonotic origins of HIV-1 and HIV-2, with the identification of SIVcpz in chimpanzees and SIVsmm in sooty mangabeys, along with their close genetic relationship to HIV-1 and HIV-2, revealing the complex evolutionary processes behind the emergence and spread of the virus in humans.

Molecular clock analyses trace the emergence of HIV-1 group M to the early 20th century, in Kinshasa, Democratic Republic of Congo, with archived samples from 1959 to 1960 providing definitive evidence of the presence of the virus before AIDS was officially identified. Phylogenetic studies reveal how viral spread occurred through transport networks, migration, and social changes, particularly in Central Africa, leading to global dissemination linked to international travel, urbanization, and socio-economic transformations. The virus spread from Africa to the Caribbean in the 1960s and later to the US and Europe, underscoring the crucial role of human mobility. Subtype B now dominates the Americas and Europe, while subtype C remains the most widespread globally, particularly in southern Africa.

Wars, migration, and socio-political crises significantly impact pandemic dynamics, with conflicts in sub-Saharan Africa, Ukraine, and Nepal being associated with increased transmission due to disrupted health systems, displacement, and heightened vulnerability to sexual violence. These factors emphasize the need for tailored public health strategies in affected areas, while advances in viral sequencing, phylogenetics, and computational modeling continue to deepen understanding of HIV evolution. These insights remain crucial for developing effective vaccines, therapies, and prevention strategies, with ongoing scientific research being vital for adapting interventions to emerging viral variants and evolving socio-economic challenges that will continue to shape the global response to HIV/AIDS.

## Figures and Tables

**Table 1:** Comparative characteristics of human immunodeficiency virus Type 1 (HIV-1) Group M and Type 2 (HIV-2) Groups A and B.

Characteristic	HIV-1 (Group M)	HIV-2 (Groups A and B)
Zoonotic Origin	SIVcpz (chimpanzees) and SIVgor (gorillas)	SIVsmm (sooty mangabeys)
Global Distribution	Pandemic (global)	Endemic (primarily West Africa)
Prevalence	>95% of all HIV infections	<5% of all HIV infections
Rate of Progression to AIDS	Rapid (Typically 8–10 years without treatment)	Slow (associated with a prolonged asymptomatic phase and lower virulence)
Average Viral Load	High	Low
Transmission Efficiency	More efficient (due to high viral loads)	Less efficient
Natural ART Resistance	Generally susceptible to NNRTIs (e.g., efavirenz, nevirapine)	Naturally resistant to NNRTIs and the fusion inhibitor enfuvirtide

**Table 2:** Global epidemiology and key characteristics of major HIV-1 group M subtypes and circulating recombinant forms.

HIV-1 Group/Subtype/CRF	Approximate Global Prevalence (Within Group M)	Dominant Geographic Regions	Key Biological and Clinical Significance
Group M (Major)	>95% of all HIV-1 infections	Global (Pandemic strain)	High transmissibility, dominant cause of the AIDS pandemic.
Subtype C	≈50%	Southern and Eastern Africa, India	Most prevalent subtype globally. Linked to high heterosexual transmission and a lower genetic barrier to resistance for some NNRTIs (e.g., Nevirapine).
Subtype B	≈12%	North America, Western/Central Europe, and Australia	Dominant subtypes in Western high-income countries. Extensive data on resistance patterns.
Subtype A	≈10%	Eastern Europe, Central Asia, and East Africa	Often associated with a potentially slower progression to AIDS in some cohorts. Subtype A6/A1 may show reduced susceptibility to long-acting injectable ART (as noted in Section 6).
CRF01_AE	≈5%	Southeast Asia (e.g., Thailand, Vietnam)	Highly prevalent CRF resulting from recombination between Subtypes A and E.
CRF02_AG	≈8%	West and Central Africa (e.g., Nigeria, Cameroon)	Most common CRF across the African continent.
Group O (Outlier)	<1%	Confined to West-Central Africa (Cameroon)	Genetically divergent; poses challenges for some diagnostic tests.

## Data Availability

No new data were created or analyzed in this study. Data sharing is not applicable to this article.
